# Differential mucosal expression of Th17-related genes between the inflamed colon and ileum of patients with inflammatory bowel disease

**DOI:** 10.1186/1471-2172-11-61

**Published:** 2010-12-13

**Authors:** Sara Bogaert, Debby Laukens, Harald Peeters, Lode Melis, Kim Olievier, Nico Boon, Gust Verbruggen, Jo Vandesompele, Dirk Elewaut, Martine De Vos

**Affiliations:** 1 Department of Gastroenterology, Ghent University, Ghent, Belgium; 2 Department of Rheumatology, Ghent University, Ghent, Belgium; 3 The Laboratory of Microbial Ecology and Technology (LabMET), Ghent University, Ghent, Belgium; 4 Center for Medical Genetics, Ghent University, Ghent, Belgium; 5 Biogazelle, Ghent, Belgium

## Abstract

**Background:**

Immunological and genetic findings implicate Th17 effector cytokines in the pathogenesis of inflammatory bowel disease (IBD). Expression of Th17 pathway-associated genes is mainly studied in colonic disease. The present study assessed the mRNA expression levels of Th17 effector cytokines (*IL17A*, *IL17F*, *IL21*, *IL22 *and *IL26*) and genes involved in differentiation (*IL6*, *IL1B*, *TGFB1*, *IL23A *and *STAT3*) and recruitment of Th17 cells (*CCR6 *and *CCL20*) by quantitative real-time PCR analysis of colonic and ileal biopsies from 22 healthy control subjects, 26 patients with Crohn's disease (CD) and 12 patients with ulcerative colitis (UC). Inflammation was quantified by measuring expression of the inflammatory mediators *IL8 *and *TNF*.

**Results:**

Evaluation of mRNA expression levels in colonic and ileal control samples revealed that *TNF*, *TGFB1*, *STAT3 *and *CCR6 *were expressed at higher levels in the ileum than in the colon. Expression of all the Th17 pathway-associated genes was increased in inflamed colonic samples. The increased expression of these genes was predominantly observed in samples from UC patients and was associated with more intense inflammation. Although increased expression of *IL17A*, *IL17F*, *IL21 *and *IL26 *was detected in inflamed ileal samples, expression of the indispensable Th17 cell differentiation factors *TGFB1 *and *IL23A*, the signaling molecule *STAT3 *and the Th17 recruitment factors *CCR6 *and *CCL20 *were unchanged.

**Conclusions:**

Our findings suggest that immune regulation is different in colonic and ileal disease, which might have important consequences for therapeutic intervention.

## Background

The location and pattern of inflammation in inflammatory bowel disease (IBD) are variable. Whereas ulcerative colitis (UC) is limited to the colon with a sharp delineation between the involved and non-involved mucosa, Crohn's disease (CD) can affect any part of the gastrointestinal tract and is associated with patchy distribution of mucosal lesions. Ileal localization occurs in about 80% of CD patients, and about 30% of CD patients have isolated ileal disease. Although it is generally accepted that IBD develops as a result of an altered immune response to luminal content in a genetically susceptible host, the mechanism by which the site of disease is selected remains unknown. Important differences in function, architecture and bacterial distribution between the ileum and colon have been described. Peyer's patches, which consist of aggregated lymphoid tissue and play a central role in the induction of mucosal immune responses, are a hallmark of the terminal ileum. Increased numbers of mucosa-associated *E. coli *are observed in IBD, and adherent invasive *E. coli *(AIEC) strains were highly associated with the ileal mucosa in CD patients. Moreover, the reduced number of goblet cells in the ileum results in decreased mucus secretion and increased contact between the mucosa and luminal content [1-3].

Several subsets of T helper (Th) cells contribute to defensive responses at inflammatory sites [4]. Dendritic cell-derived cytokines skew the differentiation of naïve CD4^+ ^T cells into Th1, Th2, Th17 or regulatory T cell (Treg) subsets. For many years, CD was believed to be mediated by Th1 cytokines, while UC was believed to be mediated by Th2 cytokines; however, recent data have implicated Th17 cells in the pathogenesis of IBD [5-9]. The invasion of extracellular bacteria into the intestinal mucosa triggers the expression of IL-23A, driving Th17 cells to release IL-17A, IL-17F, IL-21, IL-22 and IL-26, which in turn exert a number of proinflammatory effects on intestinal epithelial cells, endothelial cells, macrophages and fibroblasts [10]. In addition to their proinflammatory functions, IL-17A, IL-17F and IL-22 have been reported to induce increased expression of epithelial barrier protective genes such as defensins, mucins, tight junction proteins and lipopolysaccharide-binding proteins [11-14].

The differentiation of Th17 cells depends on the activation of janus kinase 2 (JAK2), signal transducer and activator of transcription 3 (STAT3) and the transcription factor RAR-related organ receptor C2 (RORC2) and is regulated by a combination of cytokines, including IL-6, IL-1B (IL-1β), TGFB1 (TGFβ), IL-23A, and the autocrine activity of IL-21 [4,15-20]. Chemokine (C-C motif) receptor 6 (CCR6) which is expressed on the surface of Th17 cells, contributes to their recruitment to chemokine (C-C motif) ligand 20 (CCL20) produced at the inflamed mucosa [21].

The important role of Th17 cells in the pathogenesis of IBD is also supported by genome-wide association studies, which have demonstrated that *CCR6*, *STAT3*, *JAK2*, *IL23R *and *IL12B *are CD susceptibility genes [22-24]. Interestingly, single nucleotide polymorphisms (SNPs) within *IL23R*, *IL12B*, *STAT3*, *JAK2*, the *IL22/IL26 *and the *IL2/IL21 *gene cluster have also been found to be associated with UC [22,24-27].

Although the expression of Th17-related genes has been studied previously, most studies included only colonic samples and were focused on a limited number of genes. Increased expression of *IL17A*, *IL17F*, *IL22*, *IL26*, *IL21*, *CCL20 *and *CCR6 *has been found in inflamed colonic tissues of IBD patients [4,14,28-33]. In only one study, *IL17A *and *IL23 *were mildly increased in active ileal CD samples [34].

To examine the possible differences in the expression levels of genes involved in the Th17 pathway, we assessed the mRNA levels of the Th17 effector cytokines and genes involved in the differentiation and recruitment of Th17 cells in both colonic and ileal biopsies of healthy controls, UC patients and CD patients.

## Results

### The mRNA expression levels of inflammatory cytokines and Th17-related genes in colonic and ileal samples of healthy controls

**Figure 1:**
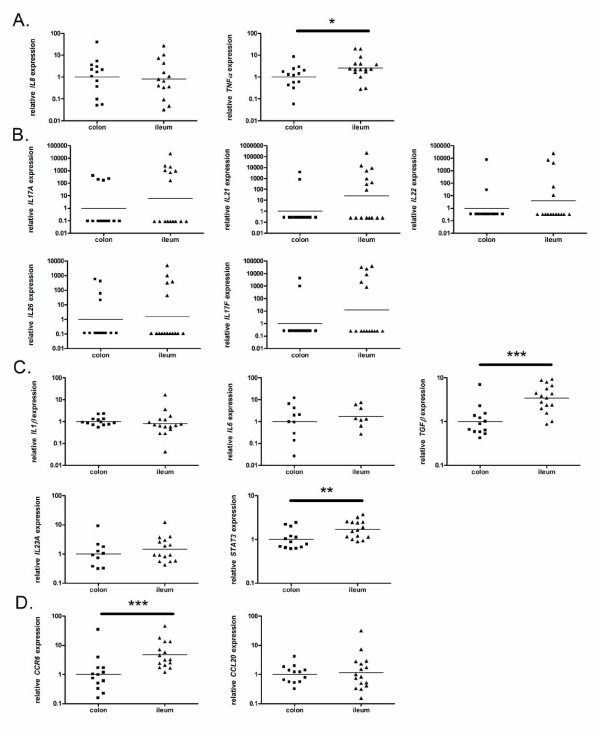
**mRNA expression levels of proinflammatory and Th17-pathway-associated genes in colon and ileum of healthy controls**. The relative transcription levels are determined by SYBR Green qPCR using the geometric mean of *GAPDH*, *HPRT *and *SDHA *as an endogenous control in colonic and ileal samples from healthy controls. The data are expressed as geometric means. The geometric mean of the colonic controls was set as 1. Data are presented on a log scale. (a) proinflammatory cytokines, (b) Th17 effector cytokines, (c) Th17 differentiation factors, (d) Th17 recruitment factors. *P < 0.05, **P < 0.01, ***P < 0.001

The expression level of the proinflammatory cytokine *IL8 *was equal in colonic and ileal controls, while the expression level of *TNF *(*TNFα*) was slightly higher in ileal control samples than in colonic control samples (P = 0.037) (Figure 1A).

The expression levels of the Th17 effector cytokines *IL17A*, *IL17F*, *IL21*, *IL22 *and *IL26 *were comparable between colonic and ileal control samples (Figure 1B); however, the expression of these cytokines in each individual sample was not consistent. In one ileal sample and none of the colonic samples, all five cytokines were expressed. In one colonic sample and four ileal samples, three to four cytokines were expressed, while in eight colonic samples and seven ileal samples, one to two cytokines were expressed. In four colonic samples and three ileal samples, none of the five cytokines were detected.

We next examined the expression of genes involved in the differentiation of Th17 cells. *TGFβ *(P = 0.0005) and *STAT3 *(P = 0.007) were expressed at higher levels in ileal control samples than in colonic control samples, while *IL6*, *IL1β *and *IL23A *expression levels were similar in colonic and ileal control samples (Figure 1C).

Expression of genes involved in the recruitment of Th17 cells was also assessed. *CCR6 *expression was higher in ileal control samples than in colonic control samples (P = 0.0008), while *CCL20 *expression was similar between colonic and ileal controls (Figure 1D).

### The mRNA expression levels of inflammatory mediators in inflamed colonic and ileal samples of IBD patients

**Figure 2:**
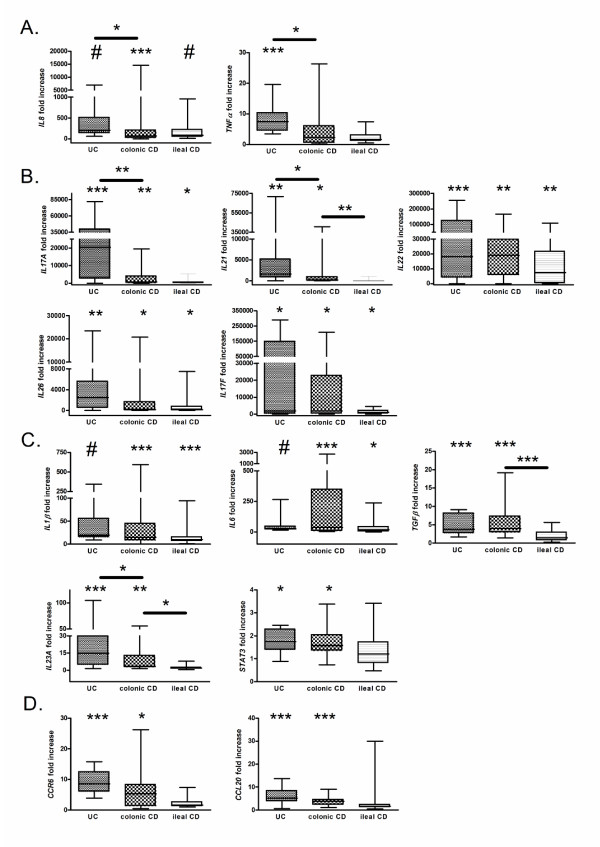
**mRNA expression levels of proinflammatory and Th17-pathway-associated genes in inflamed colonic and ileal samples of IBD patients**. Fold changes in transcription levels are determined by SYBR Green qPCR using the geometric mean of *GAPDH*, *HPRT *and *SDHA *as an endogenous control in inflamed colonic UC, colonic CD and ileal CD samples. The fold changes shown are relative to control samples. The data are presented as box plots with medians and interquartile ranges. (a) proinflammatory cytokines, (b) Th17 effector cytokines, (c) Th17 differentiation factors, (d) Th17 recruitment factors. *P < 0.05, **P < 0.01, ***P < 0.001, ^#^P < 0.0001

Although all samples were taken from endoscopically inflamed mucosa, we quantified inflammation by measuring the expression of the proinflammatory cytokines *IL8 *and *TNFα*. The expression level of *IL8 *has been shown to be associated with the grade of inflammation [35,36]. *IL8 *was strongly induced in inflamed colonic samples from UC (P < 0.0001) and CD patients (P = 0.0004) and in inflamed ileal samples from CD patients (P < 0.0001) (Figure 2A). The expression level of *IL8 *was significantly higher in UC samples than in colonic CD samples (P = 0.017). Expression of *TNFα *was only significantly increased in UC samples (P = 0.0002); however, a tendency for increase was observed in ileal CD samples (P = 0.052) (Figure 2A). Expression levels of *TNF*α were significantly higher in UC samples than in colonic CD samples (P = 0.028).

### The mRNA expression levels of Th17 effector cytokines in inflamed colonic and ileal samples of IBD patients

In UC samples, *IL17A *(P = 0.0002), *IL21 *(P = 0.0021), *IL22 *(P = 0.0003) and *IL26 *(P = 0.0017) were strongly induced, and *IL17F *(P = 0.046) was weakly induced, while in colonic CD samples, *IL17A *(P = 0.0084) and *IL22 *(P = 0.001) were strongly induced, and *IL21 *(P = 0.047), *IL26 *(P = 0.048) and *IL17F *(P = 0.011) were weakly induced (Figure 2B). In ileal CD samples, *IL22 *(P = 0.0015) was strongly induced, while *IL17A *(P = 0.013), *IL26 *(P = 0.029) and *IL17F *(P = 0.019) were weakly induced, and expression levels of *IL21 *were similar to those in ileal controls. Furthermore, in the colon, expression levels of *IL17A *(P = 0.005) and *IL21 *(P = 0.036) were significantly higher in UC samples than in CD samples. In CD samples, expression of *IL21 *(P = 0.0097) was significantly higher in the colon than in the ileum.

### The mRNA expression levels of genes involved in the differentiation of Th17 cells in inflamed colonic and ileal samples of IBD patients

*IL1β *(UC, P≤0.0001; CD, P = 0.0009), *IL6 *(UC, P≤0.0001; CD, P = 0.0007), *TGFβ *(UC, P = 0.0002; CD, P = 0.0001) and *IL23A *(UC, P = 0.0006; CD, P = 0.003) were strongly induced in both UC and colonic CD (Figure 2C). *STAT3 *was only slightly induced in colonic CD (P = 0.015) and UC (P = 0.018). Although a strong induction of the Th17 differentiation factor *IL1β *(P = 0.0007) and a weak induction of *IL6 *(P = 0.015) were observed in ileal samples, *TGFβ*, *IL23A *and *STAT3 *were not induced. In the colon, *IL23A *was expressed at higher levels in UC samples than in CD samples (P = 0.027). In CD samples, *TGFβ *(P = 0.0008) and *IL23A *(P = 0.010) were expressed at higher levels in the colon than in the ileum.

### The mRNA expression levels of genes involved in the recruitment of Th17 cells in inflamed colonic and ileal samples of IBD patients

Expression levels of both *CCR6 *(UC, P = 0.0004; CD, P = 0.011) and *CCL20 *(UC; P = 0.0003, CD; P = 0.0009) were significantly increased in colonic CD and UC samples but not in ileal CD samples (Figure 2D).

### The mRNA expression levels of the master transcription factor RORC in inflamed colonic and ileal samples of IBD patients

Two sets of primers for *RORC *were developed. The *RORC *primers detect both the full-length transcript and the shorter T cell-specific isoform, while the *RORC1 *primers detect only the full-length transcript. A strong correlation was found between *RORC1 *and *RORC *expression levels (R = 0.776, P < 0.001). When comparing the *RORC1 *and *RORC *levels between healthy controls and UC and CD patients, higher significance levels were achieved using the primer detecting both transcripts. This suggests a stronger role for the T cell-specific *RORC2 *isoform.

**Figure 3:**
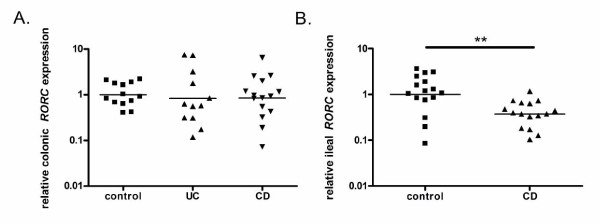
**mRNA expression levels of *RORC *in inflamed colonic and ileal samples of IBD patients**. The relative transcription levels of *RORC *are determined by SYBR Green qPCR using the geometric mean of *GAPDH*, *HPRT *and *SDHA *as an endogenous control in (a) colonic and (b) ileal samples from healthy controls, UC patients and CD patients. The geometric mean of the control samples was set as 1. Data are presented on a log scale. **P < 0.01

In colonic samples from CD and UC patients, *RORC *mRNA expression levels were comparable to those in healthy controls, while in ileal CD samples, expression levels of *RORC *were lower than those in control samples (P = 0.0019) (Figure 3).

## Discussion

A growing body of human studies and studies in mouse models has shown that Th17 effector cytokines promote chronic intestinal inflammation through the induction of multiple proinflammatory mediators. The role of Th17 cells has predominantly been studied in CD; these cells have rarely been studied in UC and have never been studied in healthy controls. Although it is generally accepted that IBD develops as a result of an altered immune response to luminal content in a genetically susceptible host, the factors influencing the selection of the disease site remain unknown. At present, even the mechanisms controlling development of ileitis and/or colitis in transgenic or gene-targeted mouse models are unclear.

In the present screening study the mRNA expression signatures of Th17 pathway-associated genes were evaluated in colonic and ileal samples of healthy controls, UC and CD patients. We first compared the expression levels in healthy colons to those in healthy ilea. Evaluation of the Th17 effector cytokines revealed no significant differences in expression levels between the colon and the ileum. Notably, these cytokines were not detectable in the majority of control samples, although expression was more frequently observed in ileal samples than in colonic samples. In addition, the expression of one Th17 effector cytokine was not necessarily linked to the expression of the other effector cytokines in a single mucosal specimen. The relative stasis of luminal content in the terminal ileum supports our observation that Th17 effector cytokines were more often present in the ileum than in the colon because IL-17A, IL-17F and IL-22 enhance the production of antimicrobial peptides, which protect the intestinal mucosa against bacterial invasion [11-14]. The diversity of bacteria present among individuals might explain why not all individuals express Th17 effector cytokines.

Recently, a subset of CD4^+ ^T cells that provide help to B cells for antibody production in germinal centers (GC), termed follicular helper T cells (Tfh), were identified [37,38]. Tfh cells produce IL-21, which is necessary for GC formation. The increased number of ileal controls expressing *IL21 *could reflect the presence of Peyer's patches in the terminal ileum, where B lymphocytes predominate in the GC. The associated increased ileal expression of the downstream signaling molecule *STAT3 *supports this assumption.

CCR6, which is present not only on Th17 cells but also on Treg cells, B cells, neutrophils and immature dendritic cells, plays a critical role in the migration of these cells to its ligand CCL20, which is produced at inflammatory sites [14,20,33]. CCR6-positive cells have been detected in lymphoid organs like Peyer's patches and isolated lymphoid follicles and seem to be more common in the ileum than in the colon. *TGFβ *was shown to be the main factor for induction of *CCR6 *mRNA expression in Th17 cells and dendritic cells [39,40]. In our study, the increased expression of *CCR6 *in ileal controls was correlated with increased ileal *TGFβ *(r = 0.785, P = 0.0003).

The increased expression of the proinflammatory cytokine *IL8 *in the inflamed colonic and ileal IBD samples confirmed the endoscopic inflammatory state of our samples. The relationship between the expression of proinflammatory cytokines and the grade of inflammation or disease activity index has been described before and is supported by our observation that *IL8 *expression levels in samples from patients in remission are similar to those of healthy controls (data not shown) [35,36]. TNFα is less useful as parameter of inflammation because reports about its expression and secretion in inflamed samples are contradictory [41-43]. In support of this view, expression of *TNFα *was increased in UC patients and not increased in CD patients.

Except for ileal *IL21*, significant induction of all Th17 effector cytokines was observed in inflamed colonic and ileal IBD samples. Moreover, induction of *IL17A *and *IL21 *was significantly more pronounced in UC than in colonic CD, and this induction was associated with more intense inflammation as defined by increased induction of *IL8*. In contrast, this association was not found in ileal CD where only marginal induction of Th17 cytokines was detected in ileal CD samples with *IL8 *expression levels similar to those observed in UC samples. Marked induction of *IL1β*, *IL6*, *TGFβ *and *IL23A*, genes involved in the differentiation of Th17 cells, was observed in colonic inflammation. The downstream signaling molecule *STAT3 *was only moderately increased in colonic samples. In ileal CD samples, except for a strong induction of *IL1β*, only a weak induction of *IL6 *and no increase in *TGFβ*, *IL23A *and *STAT3 *was detected. In parallel, significant increases in *CCR6 *and *CCL20*, genes involved in the recruitment of Th17 cells, were only observed in colonic samples, supporting a less pronounced infiltration of Th17 cells in ileal inflammation.

The observed increase in expression levels of *IL17A*, *IL17F*, *IL22 *and *IL26 *and the downstream proinflammatory cytokines *IL6 *and *IL1β *in the inflamed ileum could originate from cells other than Th17 cells. Lymphoid tissue inducer-like cells, which are important in the development of lymphoid organs, are an innate source of IL-17A and IL-22 [44]. Paneth cells, which are common in the ileum, also express IL-17A [45]. Natural killer cells, natural killer T cells and a newly identified T helper cell, Th22 cells, which are involved in inflammatory skin disorders, are sources of IL-22 [46]. Importantly, IL-17A, IL-17F and IL-22 mediate protective effects through the induction of defensins [11-14].

Although mRNA expression levels are not the optimal way to study the activity of transcription factors, the significantly reduced expression of ileal *RORC *support a defect in the Th17 pathway in ileal disease.

We should consider the fact that gene expression could be affected by the use of anti-inflammatory drugs. Although 42% of the included patients were on medication, statistical analysis did not show an effect.

Given that SNPs within *CCR6*, *STAT3*, *JAK2*, *IL23R*, *IL12B*, the *IL22/IL26 *and the *IL2/IL21 *gene cluster have been found to be associated with CD and or UC, we should take in mind the influence of these SNPs on Th17 cytokine profiles. Recently, response to anti-TNF therapy was demonstrated to be modulated by *IL23R *variants, linking Th17 function to biologicals [47]. Twenty-two percent of the samples in this study were included in the GWAS of Barrett and thus genotyped for *CCR6*, *STAT3*, *JAK2*, *IL23R *and *IL12B *[23]. Unfortunately, due to a low frequency of patients heterozygous or homozygous for the different risk alleles, such comparisons were not conclusive [47].

A lot of factors inherent to the heterogeneous nature of biopsies may influence RNA levels. Although we have to be cautious with the extrapolation of mRNA expression data to functional immunological conclusions, biological replicates and the previously demonstrated association between RNA and protein levels of most genes included in this study contribute to reliable conclusions.

## Conclusions

In conclusion, this study demonstrated important differences in the expression of Th17-associated genes in colonic and ileal disease. Inflammation of the colon of both CD and UC patients is clearly associated with increased expression levels of Th17 effector cytokines and genes involved in the differentiation, amplification and recruitment of Th17 cells, whereas in the inflamed ileum, essential factors for differentiation and recruitment of Th17 cells are missing. The differential expression of Th17-related genes between the colon and ileum could reflect different immune regulation in the colon and ileum, suggesting different therapeutic approaches for CD patients with colonic versus ileal disease. Future clinical trials of agents blocking Th17-related genes should take into account the disease location in CD patients. In addition, the difference in expression profiles between the colon and ileum also provide the potential of identifying diagnostic biomarkers.

## Methods

### Patients and samples

A total of 72 macrodissected intestinal tissue samples from 22 healthy controls, 12 UC patients and 26 CD patients were obtained during colonoscopy with a Single-Use Biopsy Forceps Radial Jaw3 (Boston Scientific, El Coyol, Costa Rica) (Table 1). The size of a biopsy specimen was usually between 2-4 mm^2 ^with an estimated average weight of 6.4 mg. UC and CD patients were diagnosed based on clinical, endoscopic and histological criteria. Patients characteristics, medication intake and the Montreal classification which gives an overview of the subclassification of IBD patients is shown in Table 1[48]. Mucosal inflammation was defined as the presence of endoscopic signs of disease activity. Samples from healthy controls were taken from the ileum and sigmoid of patients who underwent colonoscopy to screen for cancer or polyps. All biopsies collected during colonoscopy were immediately stored in RNALater (Ambion, Cambridgeshire, UK) at -80°C. The study was in accordance with the guidelines of the Helsinki Declaration (1964 and amended in 1975, 1983, 1989, 1996 and 2000) of the World Medical Association. Informed consent was obtained from all patients, and the protocol was approved by the local Ethics Committee of Ghent University Hospital (EC UZG 2004/242).

**Table 1:** Characteristics of control subjects and patients

	Colonic biopsies			Ileal biopsies	
	**Healthy controls**	**UC** **inflamed**	**CD** **inflamed**	**Healthy controls**	**CD** **inflamed**

N (Biopsy specimens)	13	12	15	16	16
Gender (male/female)	4/9	9/3	10/5	7/9	10/6
Age, yrs (mean, range)	55 (22-69)	38 (20-79)	30 (13-64)	51 (27-69)	32 (8-59)
Age at diagnosis					
(A1, A2, A3)		1/8/3	4/9/2		5/8/3
Maximal location of disease					
(L1, L2, L3)			0/3/12		5/0/11
(E1, E2, E3)		0/8/4			
Maximal disease behavior					
(B1, B2, B3)			8/5/2(6^P^)		9/5/2(5^P^)
Medication					
No	13	2	8	16	8
5-aminosalicylates		3	1		4
Corticosteroids		0	1		1
Immunosuppressives		2	3		3
Biologicals		0	1		0
Combination		5	1		0

Age at diagnosis; *A1*: 0-16 yrs, *A2*: 16-40 yrs, *A3*: >40 yrs. Maximal location of disease; CD; *L1*: solely ileal disease, *L2*: solely colonic disease, *L3*: ileal and colonic disease, UC; *E1*: ulcerative proctitis, *E2*: left-sided UC, *E3*: pancolitis. Maximal disease behavior; *B1*: non-stricturing, non-penetrating, *B2*: stricturing, *B3*: penetrating, (X^P^): number of patients when concomitant perianal disease was present.

### RNA extraction, cDNA synthesis and amplification

Total RNA was extracted from 2-3 pooled mucosal samples using an RNeasy Mini Kit (Qiagen, Westburg BV, The Netherlands) with on-column DNAse treatment (Qiagen). Needle homogenization was performed. The total RNA was quantified using spectrophotometry (Nanodrop; Thermo Scientific, Wilmington, USA) and ranged from 150 ng to 16.8 μg with an average of 5.9 μg total RNA. The quality of the RNA, expressed as RNA quality indicator (RQI), was checked by automated electrophoresis (Experion, Bio-Rad, Hercules, California) and ranged from 7.4 to 10 with an average of 8.6. Starting from 20 ng of total RNA, the WT-Ovation RNA Amplification System (Nugen Technologies Inc., San Carlos, USA) was used to the letter of the manufacturer's instructions, generating approximately 6 μg of cDNA. First strand cDNA is prepared from total RNA using both oligo-dT and random hexamer primers and reverse transcriptase. After the generation of double strand cDNA, a DNA amplification step developed by NuGEN was performed. The cDNA was diluted to 50 μl.

### Quantitative real-time PCR

PCR amplification reactions were carried out in a total volume of 8 μl containing 2× SYBR Green I Master Mix (Eurogentec, Seraing, Belgium), 3 μl 1/100 cDNA (~3.75 ng) and 250 nM forward and reverse primers (BioLegio, Nijmegen, The Netherlands). All reactions were performed in 384-well plates (LightCycler 480 Multiwell Plates 384, white and LightCycler 480 Sealing Foils from Roche) on the CFX384 real-time PCR detection system (Bio-Rad, Hercules, California), followed by a regression Cq value determination method. Cycling conditions were as follows: 95°C for 10 min followed by 45 cycles of 95°C for 10 s and 60°C for 30 s, followed by a dissociation curve analysis from 60 to 95°C. Because instrument and liquid handling variations were shown to be minimal using the Tecan Freedom Evo robot for pippeting (< 6% CV in 0.5 μl and <3% CV in 2 μl), and a large number of biological replicates were used, no PCR replicates were carried out. Primers containing neither SNPs nor secondary structures were designed for Glyceraldehyde 3-phosphate dehydrogenase (*GAPDH*), Succinate dehydrogenase complex subunit A (*SDHA*), Hypoxanthine-guanine phosphoribosyltransferase (*HPRT*), *IL8*, *TNF*, *TGFB1*, *IL1B*, *IL6*, *IL23A*, *CCR6*, *STAT3*, *IL17A*, *IL17F*, *IL21*, *IL22*, *IL26*, *CCL20 *and *RORC *(Table 2). For *RORC*, two sets of primers were designed, *RORC*, which detects the mRNA levels of both isoforms, and *RORC1 *which detects only the full-length transcript. BLAST searches confirmed that only the target genes were 100% covered. A 6 point 4-fold standard dilution series (highest concentration; 32 ng/μl) of a cDNA mixture of all samples included in the study diluted in 5 ng/μl tRNA (Roche) was used to test the PCR efficiency of the primers. The dynamic range had to cover at least 3 orders of dilution. Only primers with an efficiency between 88% and 112% were retained (Table 2). Correlation coefficients of the targets were between 0.9843<R^2 ^< 1, with a mean of 0.9942 (Table 2). The PCR efficiency for each gene was calculated according to the equation E = 10 (-1/slope). Each sample has been revised for a melting-curve with a single sharp peak with a high correlation between the observed and the expected Tm (mean variation of 0.9°C). Samples with other patterns than a single sharp peak at the expected Tm, defined as multiple peaks, a single broader peak or a shoulder peak, were omitted. Cq values of samples with flattened melting-curves were set as 45. An amplification signal in the no template control (NTC) was ignored as long as the difference in Cq value between the NTC and the highest Cq >5. Although the pre-amplification method of NuGEN does not amplify genomic DNA, possible gDNA contamination was assessed using intronic primers. We confirmed that gDNA was undetectable in a dilution of up to 32 ng/μl cDNA [49]. The mRNA expression level of each gene was determined in Excel by using the comparative 2-(delta delta Cq) method and normalized to the geometric mean of the stably-expressed reference genes *GAPDH*, *SDHA *and *HPRT *as determined by geNorm [50].

**Table 2:** Sequences and exon locations of qPCR primers, amplicon lengths, PCR efficiencies and correlation coefficients

Gene Symbol	Reference sequence	Forward primer	location	Reverse primer	location	Amplicon length (bp)	PCR efficiency (%)	**R**^**2**^
*GAPDH*	NM_002046	TGCACCACCAACTGCTTA C	Ex7	GGCATGGACTGTGGTCATGAG	Ex7-8	87	109	0,99
*SDHA*	NM_004168	TGGGAACAAGAGGGCATCTG	Ex2	CCACCACTGCATCAAATTCATG	Ex3	86	104	0,9914
*HPRT*	NM_000194	TGACACTGGCAAAACAATGCA	Ex5-6	GGTCCTTTTCACCAGCAAGCT	Ex6	94	110	0,9985
*IL8*	NM_000584	GAATGGGTTTGCTAGAATGTGATA	Ex4	CAGACTAGGGTTGCCAGATTTAAC	Ex4	129	99	0,9995
*TNF*	NM_000594	CCTGCCCCAATCCCTTTATT	Ex4	CCCTAAGCCCCCAATTCTCT	Ex4	80	88	0,9991
*IL17A*	NM_002190	CCATAGTGAAGGCAGGAATC	Ex3	CGGTTATGGATGTTCAGGTT	Ex3	108	98	0,998
*IL17F*	NM_052872	AGCGCAACATGACAGTGAAG	Ex1	GTGTAATTCCAGGGGGAGGT	Ex2	105	89	0,993
*IL21*	NM_021803	ACTTGGTCCCTGAATTTCTGC	Ex1-2	TTTGTGGAAGGTGGTTTCCTC	Ex3	169	92	0,9891
*IL22*	NM_020525	TTCCAGCAGCCCTATATCACC	Ex1	GCTCACTCATACTGACTCCGTG	Ex2-3	125	102	0,9937
*IL26*	NM_018402	ATCAAAGCAGCATGGCTCAAA	Ex1	GCAGTTGACCAAAAACGTCTTCC	Ex3	154	104	0,9894
*IL1B*	NM_000576	CACGATGCACCTGTACGATCA	Ex5	GTTGCTCCATATCCTGTCCCT	Ex5	121	90	0,9991
*IL6*	NM_000600	ATTCTGCGCAGCTTTAAGGA	Ex5	AACAACAATCTGAGGTGCCC	Ex5	119	104	0,9956
*TGFB1*	NM_0006600	GGCCAGATCCTGTCCAAGC	Ex1	GTGGGTTTCCACCATTAGCAC	Ex1	201	93	0,9872
*IL23A*	NM_016584	TTCTGCTTGCAAAGGATCCA	Ex3	AATATCCGATCCTAGCAGCTTCTC	Ex3	64	106	0,9957
*STAT3*	NM_139276	GATCCAGTCCGTGGAACCAT	Ex21	ATAGCCCATGATGATTTCAGCAA	Ex21	74	106	1
*CCR6*	NM_031409	GTGCAAGTTGCTAAAAGGCATC	Ex3	CGAATGACTTAGTCGCCTGT	Ex3	112	89	1
*CCL20*	NM_004591	TGCTGTACCAAGAGTTTGCTC	Ex1	CGCACACAGACAACTTTTTCTTT	Ex3	220	98	0,9914
*RORC*	NM_005060	GTAACGCGGCCTACTCCTG	Ex4	GTCTTGACCACTGGTTCCTGT	Ex5	227	102	0,9944
*RORC* (*RORC1*)	NM_005060	GCAAAGAAGACCCACACCTC	Ex2-3	GCACCCCTCACAGGTGATAA	Ex3	102	112	0,9843

According to the MIQE guidelines, the minimum information for publication of quantitative real-time PCR experiments was provided [51].

### Statistical analysis

Statistical differences were assessed using a non-parametrical Mann-Whitney U test (two tailed probabilities). The correlations were analyzed with Spearman's correlation coefficient. *P*-values less than 0.05 were considered significant. Statistical analysis was performed using SPSS software 11.5 (SPSS, Chicago, USA).

## Authors' contributions

SB had substantial contributions to the conception, design, execution and analysis of the study, and drafted the manuscript; DL participated in the design and interpretation of the data; HP and MDV carried out the sampling of gut specimens and contributed to the interpretation of the data, LM and KO contributed to the RNA extraction, RNA quality determination, cDNA synthesis and qPCR analysis; JV participated in designing and organizing the qPCR analysis; JV, NB, GV and DE participated in critically revising the manuscript; MDV carefully revised and edited the manuscript with important intellectual contributions and coordinated the research group. All authors read and approved the final version of the manuscript.
